# Segmentation of the iliac crest from CT-data for virtual surgical planning of facial reconstruction surgery using deep learning

**DOI:** 10.1038/s41598-024-83031-0

**Published:** 2025-01-07

**Authors:** Stefan Raith, Tobias Pankert, Jônatas de Souza Nascimento, Srikrishna Jaganathan, Florian Peters, Mathias Wien, Frank Hölzle, Ali Modabber

**Affiliations:** 1 Department of Oral and Maxillofacial Surgery, RWTH Aachen University Hospital, Pauwelsstraße 30, 52074 Aachen, Germany; 2 Inzipio GmbH, Krantzstr. 7 Building 80, 52070 Aachen, Germany; 3 Institute of Imaging and Computer Vision, RWTH Aachen University, Kopernikusstraße 16, 52074 Aachen, Germany

**Keywords:** Convolutional neural networks, Deep learning, Pelvis, Segmentation, Computed tomography, Virtual surgical planning, Medical image analysis, Musculoskeletal system, Bone, Computational science, Computer science

## Abstract

Background and objectives: For the planning of surgical procedures involving the bony reconstruction of the mandible, the autologous iliac crest graft, along with the fibula graft, has become established as a preferred donor region. While computer-assisted planning methods are increasingly gaining importance, the necessary preparation of geometric data based on CT imaging remains largely a manual process. The aim of this work was to develop and test a method for the automated segmentation of the iliac crest for subsequent reconstruction planning. Methods: A total of 1,398 datasets with manual segmentations were obtained as ground truth, with a subset of 400 datasets used for training and validation of the Neural Networks and another subset of 177 datasets used solely for testing. A deep Convolutional Neural Network implemented in a 3D U-Net architecture using Tensorflow was employed to provide a pipeline for automatic segmentation. Transfer learning was applied for model training optimization. Evaluation metrics included the Dice Similarity Coefficient, Symmetrical Average Surface Distance, and a modified 95% Hausdorff Distance focusing on regions relevant for transplantation. Results: The automated segmentation achieved high accuracy, with qualitative and quantitative assessments demonstrating predictions closely aligned with ground truths. Quantitative evaluation of the correspondence yielded values for geometric agreement in the transplant-relevant area of 92% +/- 7% (Dice coefficient) and average surface deviations of 0.605 +/- 0.41 mm. In all cases, the bones were identified as contiguous objects in the correct spatial orientation. The geometries of the iliac crests were consistently and completely recognized on both sides without any gaps. Conclusions: The method was successfully used to extract the individual geometries of the iliac crest from CT data. Thus, it has the potential to serve as an essential starting point in a digitized planning process and to provide data for subsequent surgical planning. The complete automation of this step allows for efficient and reliable preparation of anatomical data for reconstructive surgeries.

## Introduction

In planning of surgical interventions for the bony reconstruction of the mandible, autologous iliac crest flaps have become an established choice alongside fibula grafts^1^. While computer-assisted planning methods are gaining increasing importance, the preparation of geometric data based on CT imaging (known as segmentation) remains a predominantly manual process.

Facial reconstructive surgery is a key component in the treatment of various maxillofacial conditions, including tumors on the maxillary bone or the tissue surrounding it^2^, as well as penetrating injuries such as caused by knives or bullets^3^. The surgery aims to address both the aesthetic and functional concerns of the patient and thus restore essential functions that were affected by the trauma, such as speech, deglutition, and motion of the lips^2^. In this process, the surgeon is responsible for restoring the affected continuity in the maxillary or mandibular bone, allowing for a proper placement of dental implant^1^.

Currently, the most effective approach is using autologous bone transplants. Besides the free fibular flap the most commonly used autologous transplant is from the iliac crest^4^. The pelvic iliac crest free flap has a particular relevance given its size, comparable to the natural mandible, effectively supporting the structure of the chin and lower section of the face. The curved structure of the bone also facilitates the reconstruction^5,6^. In comparison to the fibula flap, however, the donor site morbidity can be of a larger concern since rehabilitation is required to restore natural movement, and special attention has to be taken to restore the abdominal wall to prevent hernia^1^. Nevertheless, a comparison of morbidity between fibular and iliac crest flaps showed patients reporting only minor morbidities for the latter case, where there was a significantly larger amount of pain for the fibula free flap transplants^7^.

The planning procedure is a core part of complex surgical interventions such as mandibular reconstruction with bony transplants^8^. Recently, there has been increasing research attention on this step, recognizing that well-planned procedures positively impact the surgery as a whole^9,10^. This focus has guided the growth in the search for patient-specific planning solutions in the field of facial reconstructive surgery in recent years^11^. Consequently, the use of computer-assisted tools in surgical preparation has gained special attention, particularly in mandibular reconstruction surgeries. This is because, with the assistance of 3D modeling of the bones, a patient-specific cut guide can be developed beforehand, along with osteosynthesis plates^12^. These advancements optimize surgical procedures in ways that were not possible with freehand surgery. Al Sabahi et al. compared surgeries in patients with and without patient-specific models during the planning procedure^13^. This study also found better results in surgeries that used virtual surgery planning. In this case, it was even attested that the aesthetic outcomes were significantly better in the computer-assisted surgeries, besides other advantages.

Recently, Bertier et al. conducted a CT symmetry study to compare the accuracy of mandibular reconstruction surgeries between patients using virtual surgery planning and those employing traditional freehand methods. The findings indicate enhanced symmetry with the use of computer-assisted techniques^14^. In 2021, van Baar et al. conducted a complete systematic review of the impact of computer-assisted surgery in maxillary reconstruction, based on available literature. The review raised important criticisms regarding the non-standardization of evaluation metrics for surgeries, which differ much from each other, and therefore state different risks when comparing results in the literature. Nevertheless, the author still recognizes the impact of computer-assisted surgeries on the field, its important results achieved so far, and the future prospects for this research field^15^. Likewise, Chan et al. also synthesizes the current literature on maxillary reconstruction surgery using virtual surgical planning. Its conclusions are also similar, attesting to the inconsistencies in the evaluation metrics between different surgeries in research, but making a clearer highlight on the advantages found in the screened publications. They also make a proposition on a reporting tool for standardizing surgical outcome evaluation^16^.

In summary, in the facial reconstructive surgery context, computer-assisted techniques are proven to be more efficient and provide better results overall than traditional planning strategies, being more comfortable to the patient and the surgeon. Even though the use of virtual surgery planning has been showing great advantages in mandibular reconstruction surgery in general, the key limitation to this approach is the preparation of the 3D models to be used in the procedure^17^. The first step of this process is to acquire CT images of the region of interest and then perform a segmentation of the bone to be modeled before the surgery. This process is still the main bottleneck in the virtual surgery planning procedure because traditional segmentation techniques are highly limited in addressing complex problems such as this one, and they are especially deficient in differentiating between the bones relevant for a specific surgical procedure and others. At the same time, novel and deep learning-based techniques have only recently been studied in the mandibular reconstruction field^17^.

Different traditional techniques have been used to perform segmentations in the medical image context. For instance, Kaur et al. uses edge-detection techniques to segment fractures, brain, and lung images^18^, Sun et al. used a multi-atlas method to accurately segment brain MRI^19^ and Jafarian et al. used a variational level set-based framework to segment newborn cranial bone from CT images^20^. Challenges in these studies include the need for large data sets for creating the model and the complexity due to numerous features in the multi-atlas technique.

Focusing on pelvic bone segmentation, the central theme of this study, we discuss relevant related works. Lamecker et al. created a statistical shape model of the bone to perform the segmentation from CT data^21^ for pelvic bone segmentation. The model was created using 23 manually segmented CT volumes. The segmentation approach demonstrated encouraging outcomes, achieving an average surface distance of 1.8 mm in leave-one-out evaluations. Nonetheless, a manual initialization step is necessary, hindering full automation.

Similarly, Seim et al. used a data set of 50 pelvic CTs to create a complex 3D statistical shape model of the bone^22^. The method is slightly different from the former, having a free form segmentation step based on optimal graph searching as a last step. Also, aiming to have a fully automated method, the authors use an automatic initialization based on a 3D Hough Transform method. The results achieved in that study are also significantly better, with an average surface distance of around 0.7 mm overall and remarkably worse results only at the region of the sacrum.

However, threshold segmentation is still the most common basis for segmentation used in the context of surgery planning, as van Eijnatten et al. report in a review of segmentation techniques for additive manufacturing of anatomical models, i.e. anatomical three-dimensional printing^23^. This analysis shows that most segmentation applications used either a direct global threshold or some variation of this approach.

Various strategies have been explored to address this bottleneck in the planning process. For example, Ulbrich et al. introduced a virtual reality-based environment for conducting CT bone manual segmentation, moving away from the conventional desktop screen approach^24^. This innovation not only enhanced the learning curve and segmentation speed but also made the process less taxing for study participants, as reported. Thus, such tools bear the potential to improve the manual workload necessary in threshold-based segmentations. However, it does not eliminate them, which is thus the focus of the present work.

Deep Learning methods are achieving excellent results in the medical image domain, which can be of high relevance for a possible application in the facial reconstructive surgery context. For instance, Hemke et al. uses multiclass U-Nets to segment pelvic bone, muscles, and fat from CT-slices^25^. To achieve this, 200 images were manually segmented, and the Neural Network was trained with the help of data augmentation. Even though this study is focused on body composition measures, it already shows remarkable results regarding the segmentation of the pelvic bones, achieving Dice scores of 0.92 in a 2D segmentation.

Using multi-parametric MRI images, Liu et al. developed a framework to segment the pelvic bone with different classes^26^. This result is of extreme importance to the pelvic bone segmentation field in general because it segments the full 3D bone from volumetric data instead of the 2D segmentation proposed by Hemke et al.^25^, and achieved a DSC of 0.80 on the full bone.

Furthermore, Liu et al. used a two-staged U-Net-based pipeline to perform a multi-class segmentation on the pelvic bone using data from different datasets^27^. It divides the bone between the lumbar spine, sacrum, left hip, and right hip and uses a proposed signed distance function post-processor. The results achieved are an impressive DSC of 0.978 on the whole bone and a Hausdorff Distance of 5.5 voxels. This work is of special significance because it is the first, and so far, only, to conduct a segmentation of the whole pelvic bone from volumetric CT images, similar to the one which is aimed throughout the present work.

In previous work of our group, published by Pankert et al., we conducted similar work focusing on facial reconstructive surgery planning with an in-house developed 3D U-Net-based two-step segmentation approach to segment the mandibular bone^17^. There, we could demonstrate by application of a two-step approach instead of only one 3D U-Net significant advantages and achieved accuracies of over 0.94 on the dice similarity coefficient (DSC) and average surface distance (ASD) values lower than 0.36 mm.

Therefore, the present work intends to investigate the possibility of applying this two-step segmentation method for the first time to donor bones for an automated process pipeline in facial reconstruction with autologous iliac crest transplants. Thus, the present work aims at delivering the bony geometries as a key component for the planning procedure, which by its automation may overcome the time-consuming step of segmentation.

Thus, the goal of the presented work was to develop and test a procedure for the automated segmentation of the pelvic bone for subsequent reconstruction planning. The hypothesis of the present work is that a Convolutional Neural Network in the 3D U-Net architecture can be adapted for the task of segmentation of the iliac crest with sufficient accuracy to be used in the process chain of surgical planning of facial reconstructions with autologous iliac crest transplant.

## Materials and methods

In this study, a two-step segmentation approach is proposed to segment the bony pelvis, consisting of the ilium, the ischium and including the sacrum. This approach has shown to be beneficial in several applications on related tasks, such as mandibular segmentation^17^.

### U-Net architecture for pelvic bone segmentation

**Fig. 1:**
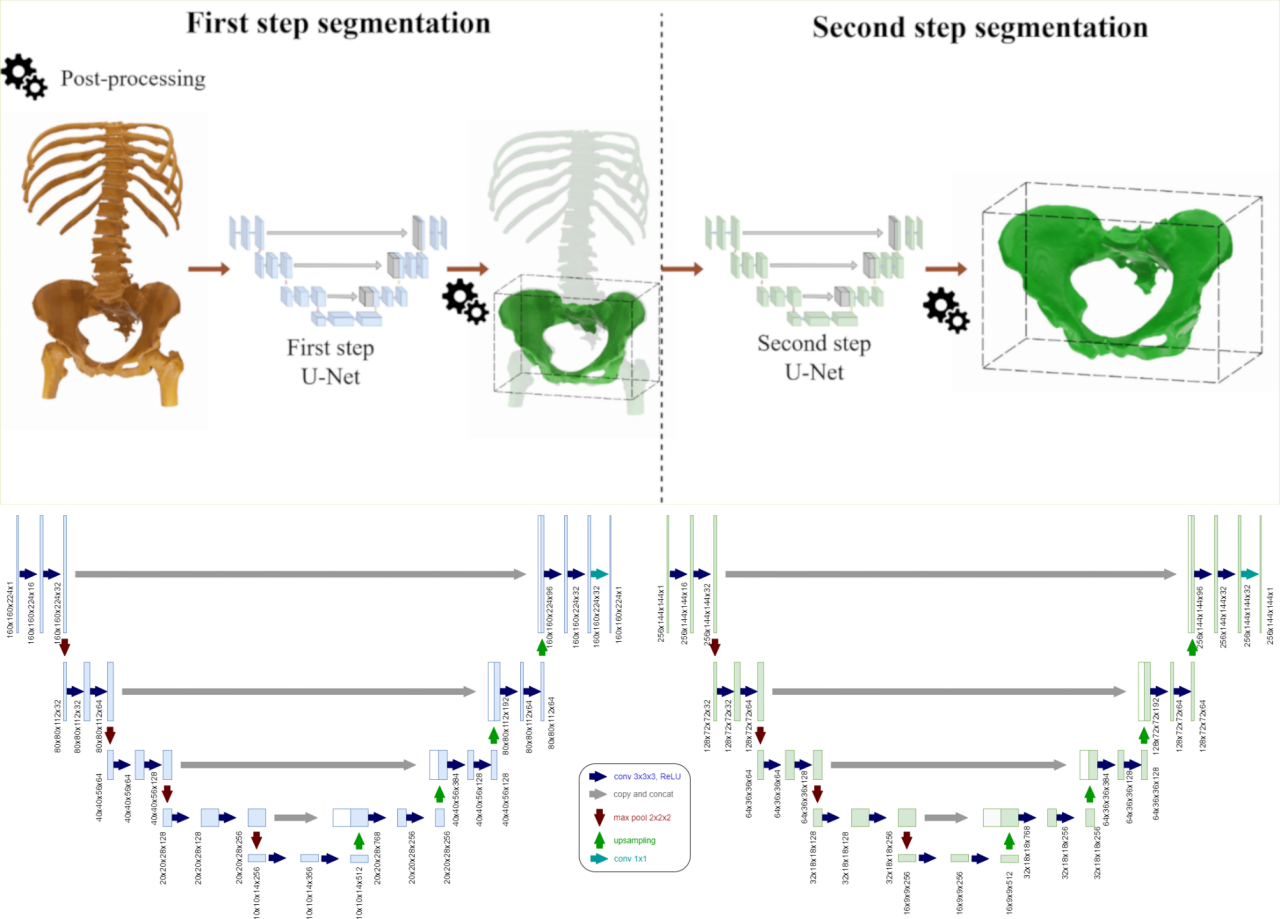
Top: First step segmentation from whole field of view (left) and second step segmentation within the cropped region of interest as defined around the first step result (right). Bottom: Detailed view of the two stages of the 3D U-Net with identical architectural layout and different resolution.

The proposed framework consists of two separately trained 3D U-Nets that both segment the pelvic bone, with the difference that the first is responsible for segmenting the bone based on the full field of view of the CT acquisition, and the second learns how to segment the bone based only on a narrower region of interest cropped around the relevant bones. A diagram showing the pelvic bone approach is found in Fig. 1. The proposed method is similar to the one implemented in^17^, with the main difference of using two different resolutions for the two steps. This modification is done to adapt the method to the particularities of this bone and the initial field of view. The resolution for the first step is chosen after considering the average amount of CT slices on the available dataset. While every slice has 512 × 512 as resolution, the pixel spacing and the number of slices vary considerably within the datasets. Based on a statistical overview of the dataset to acquire average aspect ratio, the resolution chosen for the first step was 160 × 160 × 224. Similarly, for the second step, an analysis is made on the ground truth of the pelvic bones. Succeeding a statistical analysis of the extents of all the available ground truth meshes the voxels in each dimension were chosen to remain as proportional to the average aspect ratio of the bones. The final used resolution was chosen to be 256 × 144 × 144. To bring the imaging data to the designated common spatial resolution, a scaling of the respective datasets was applied to the data before actual resampling in both steps accordingly.

### 3D U-Net architecture

The 3D U-Net, as the Convolutional Neural Network architecture used in the present experiments for segmenting the pelvic bones, is a fully Convolutional Network and can, therefore, receive any image shape as an input and return an output image with the same resolution^28,29^. This characteristic allows this architecture to perform well in the segmentation experiments presented in this work. Here, the U-Net architecture used for the experiments is a variation of the three-dimensional U-Net described in^29^. It has ten convolutional layers in the encoding path, with four max pooling layers in between. Following the standard seen in literature, the feature maps are doubled after every pooling step. The network uses convolutional kernels of 3 × 3 × 3 size and 2 × 2 × 2 sized max pooling layers. Furthermore, the decoding path consists of nine Convolutional layers and four up-sampling layers. The up-sampling factor used in the described architecture was 2 × 2 × 2, which means the output of every up-sampling layer is double its input for every dimension. An overview of the 3D U-Net described above can be seen in Fig. 1.

### Transfer learning

Transfer Learning is a technique used in the most diverse set of machine learning applications. It consists of using the acquired knowledge from one task to reduce learning costs in a different task^30^. Yosinski et al. showed that initializing weights of Deep Neural Networks based on an already trained model improves the generalization performance of the model, even for nonrelated tasks^31^. The process of training the pre-trained weights for a new task is called fine-tuning, and often specific regions of the Neural Network are responsible for learning the general characteristics of images. Therefore, they can be used for general tasks. Consequently, it is not unusual to freeze some layers of the Convolutional Neural Network during the finetuning procedure and only update a reduced set of parameters. This feature was already used in the medical field to train networks to identify COVID infection based on chest X-rays^32^, to classify brain tumor MRI images^33^, and to classify endoscopic colonoscopy images^34^. Given the successful results found in the literature, a Transfer Learning approach was also used to provide a better initialization to the Convolutional Neural Network training potentially improving the efficiency and the results of the experiments. Training models for the second step of the proposed pipeline is expected to be a less complex task since the Neural Network must only learn inside the region of interest for the bone. Thus, the relative resolution is higher than for the first step. Based on that, the method applied in this work was to utilize the weights from the second step as initialization for the first step of the training procedure. The second step network did not use any transfer learning and instead was initialized with a Glorot uniform distribution^35^.

### Training

The loss function is highly relevant for the learning procedure, being responsible for guiding the direction of the training. Therefore, the decision on the loss function to be selected for the experiment must be taken carefully, considering which can be more beneficial for the specific task. Different losses have been used for similar segmentation tasks, with the cross-entropy loss being one of the most popular ones^36^. This loss function computes the similarity between the output probabilistic distribution and the target binary distribution. Several variants of cross-entropy are also commonly used in medical segmentation tasks, including focal loss^37^, which addresses the foreground-background imbalance in segmentations, and weighted cross-entropy, defined in the original U-Net implementation, which gives different weights for different segmentation classes^28^. An important variant is the Dice loss, as previously defined by Milletari et al.^38^ based on the Dice score, measuring the volumetric overlap between two entities. This loss, therefore, penalizes the overlap mismatch and can also be generalized for K classes, being defined as LDice = 1 − DS. Considering its close relation to the actual segmentation task and experience from preliminary work, the Dice loss was chosen for the present study.

Both models were trained using the Adam optimizer^39^ with a batch size of 1 and an initial learning rate of 5e-4. The learning rate was reduced by the factor 10 after 10 epochs without loss improvement. The training was stopped after the loss not decreasing for 30 epochs. Thanks to the large corpus of available training data, we did not need to use any data augmentation.

### Post-processing

After the actual pipeline of segmentation, the binary image data at the resolution of the second stage U-Net was post-processed to allow for a better representation of the acquired data as a surface mesh using marching cubes algorithm. This stage uses the outputs of the second Neural Network and finally transforms the volumetric predictions to triangulate 3D meshes, that are afterwards used in the virtual planning of the surgical procedure. The importance of the post-processing step is to transform the binary discrete output, which differs significantly from the actual bone, into a smooth mesh, which has less deviation to the expected result^17^.

The first step of post-processing is a binary erosion of data about one layer of voxels to compensate for a dilation of the binary mask while converting ground truth meshes to volumetric masks for training. This step is significant for the subsequent smoothing, especially because it removes pixels that are only segmented because of limitations on the down-scaled resolution.

**Fig. 2:**
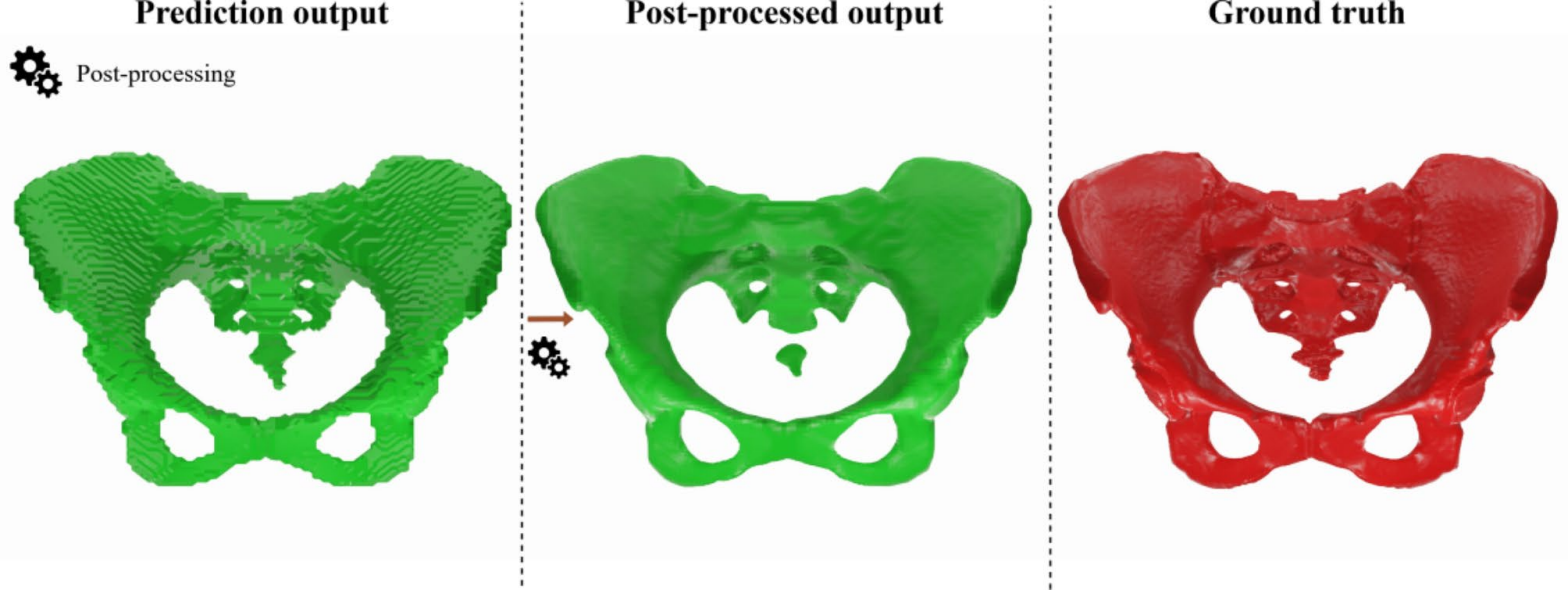
Example visualization of a triangulated raw prediction output (left), the post-processed output (middle) and corresponding ground truth segmentation (right).

After the referred erosion of the data, and before the binary image is finally transformed to a mesh, a Gaussian filter is used on the image, aiming to reduce noise and add smoothness to the data^40^. The combination of erosion and the Gaussian filter may lead to small, disconnected parts, predominantly at the sacrum (Fig. 2). However, these parts are still considered in the subsequent evaluations.

At the end of this process, the voxel representation of the segmented bone is transformed into a mesh using the marching cubes algorithm^41^. This algorithm classifies vertices as true or false based on a given iso-value as a threshold. Subsequently, the target surface is covered using a look-up table. The post-processing step is of high importance for the proposed framework and is responsible for transforming the binary output of the Neural Network into a 3D model that has the smoothness of the real bone (Fig. 2).

### Preparation of medical imaging data

Overall, a collective of 1398 CT scans could be accessed from the PACS (picture archiving and communication system) of RWTH Aachen University Hospital, along with correspondent manual segmentation that was considered to be the ground truths for the automated segmentation task, in order to be used for the present study. The set of data was used in a previous study focusing on the anatomic variability of the iliac crest in relation to different age and sex groups as well as its potential applicability in the process of surgical planning of transplant surgery to bridge defects in the facial skeleton. We confirm that all methods were carried out in accordance with relevant guidelines and regulations and an institutional approval (EK 260/20) of the Independent Ethics Committee of the Faculty of Medicine of RWTH Aachen University Hospital was obtained. Due to the retrospective nature of the study, the Independent Ethics Committee of the Faculty of Medicine of RWTH Aachen University Hospital waived the need of obtaining informed consent.

The raw medical imaging datasets analyzed during this study are not publicly available due to privacy concerns, as per the guidelines of the ethics committee, which permits access to the original data only for a specific group of named researchers.

**Fig. 3:**
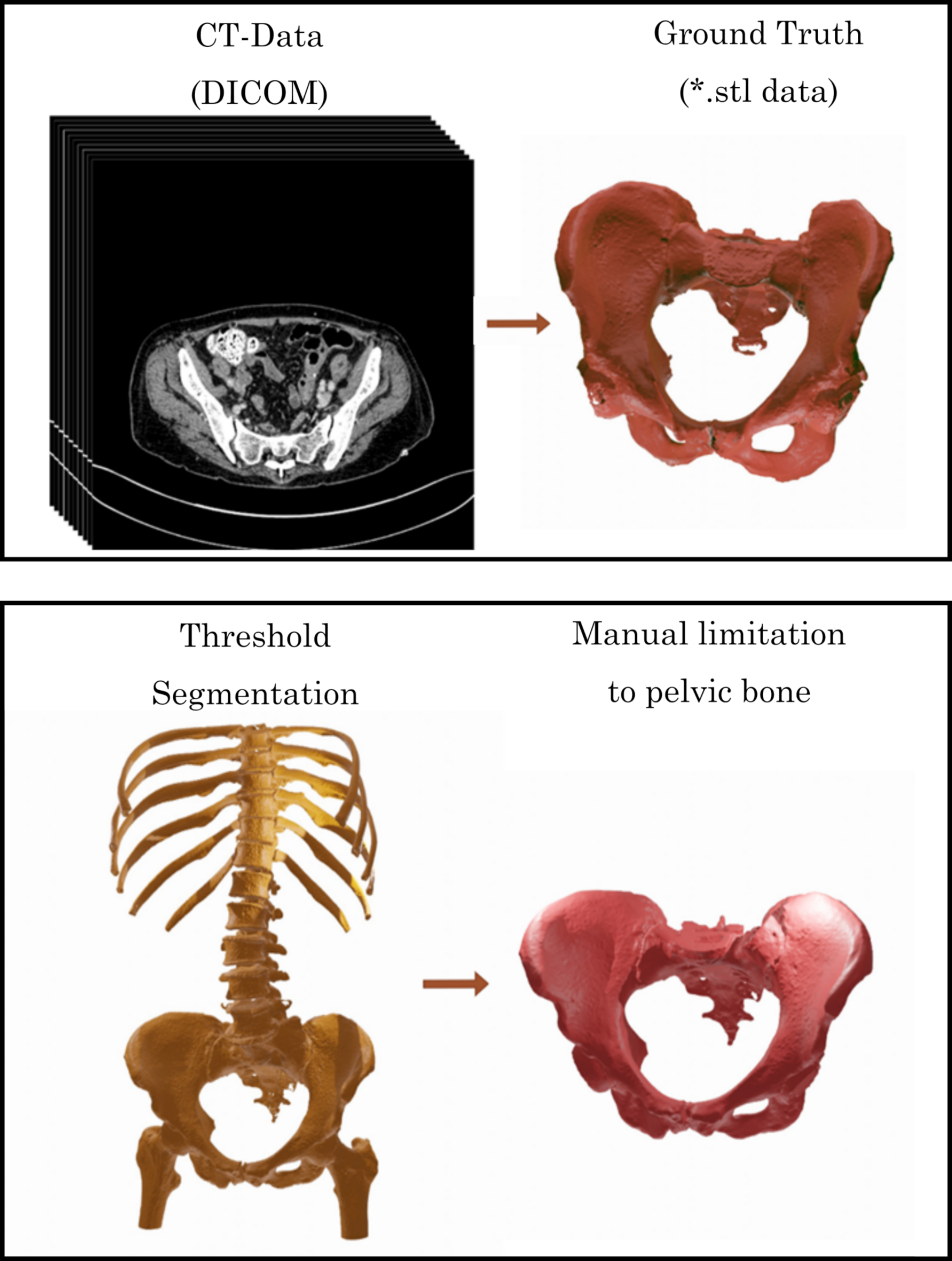
Example CT scan data (top left) and corresponding manually generated ground truth surfaces model (top right). Threshold segmentation based on the whole field of view and corresponding ground truth of that set of data (bottom).

Segmentation was done manually by one experienced expert with the software Mimics 14.0 (Materialise, Leuven, Belgium) in order to capture the surface of the pelvic bones with special interest on a careful representation of the iliac crest, as this is the most relevant part for transplantation. Correspondingly, lower priority was put on the sacrum and on the femoral cavities, where the connection to the spine and the femoral heads was segmented with less demands on accuracy. Hence, this prerequisite needs to be considered in any evaluation of these specific parts when looking at the whole pelvic bone (Fig. 3).

The dataset used in this work covers a wide range of ages and even distribution of sexes. However, the data quality varied significantly within the dataset. Not all CT scans covered the entire pelvic bone, for some, the bottom part of the bone was cropped at the caudal sides. In addition, some patients had metal implants, either on the pelvic bone joints or in other regions around the bone. These implants significantly affect image quality and could potentially impact the training procedure of the models subsequently.

Other problems were found on the data set: Some images for instance were acquired for patients tilted on the bench, which causes the images to be completely different in orientation from the vast majority. Finally, some images also had a remarkably lower quality than the average.

To acquire an overview of the available data and, thus, to evaluate the exact number of patients that have each of the problems described, threshold segmentation images were generated for all 1398 CT scans available. Subsequently, visual investigation was done to individually analyze the data, and all problematic cases were labeled accordingly and excluded from the subsequent study protocol.

Since the amount of data available was excessive and above the conventional limit of comparable studies using machine learning tasks, massive compared to the previous work, a possible exclusion of the CT images that had the related problems would not impact negatively. In fact, this removal is expected to be positive as there is no interest in making the Neural Networks to be able to deal with implants or tilted patients and having this data in the training could only disturb them in the end. Therefore, after the last step, a decision was made to remove all data that did not follow the desired standard of the dataset. Following this convention, 394 datasets were excluded, as patients had either implants, or were tilted, or parts of the bone were cropped, or it had considerably worse quality than the standard. After application of these exclusion criteria, data from 1004 patients was available, all of sufficient to excellent quality and ready to be used in the subsequent experiments. Out of this data a randomly selected subset of 400 patients was chosen for training and validation (training *n* = 360, validation *n* = 40) for most efficient use of GPU memory. For the test set, a collection of 177 different patients was reserved and statistically evaluated as described below.

For the use of these segmented meshes as ground truth data for the training pipeline, a reverse calculation of a volumetric binary mask in the resolution of the original CT was required. The algorithm for doing so was implemented by means of the trimesh library in python and considered all voxels that have some extent inside the triangulated mesh as belonging to the segmentation mask, thus generating a greedy mask that contains the full triangulated mesh.

###  Statistical evaluation

The collective of *n* = 177 data sets in the test collective were evaluated by three different metrics. These are namely (1) Dice similarity coefficient (DSC), (2) symmetrical average surface distance and (3) Hausdorff-Distance with its modification to show the 95% percentile in a symmetric calculation. The metrics were all computed between the triangulated data of ground truth meshes and the post-processed output meshes of the second segmentation stage. The decision to use that representation of data rather than a voxel-based comparison was chosen as this output is relevant for further processing of the geometrical data in surgical planning.

As it is known that the so-called ground truth data had limited accuracy in regions that are not relevant for transplantation (see explanation above), any evaluation of accuracy on the whole bone data have limited value.

**Fig. 4:**
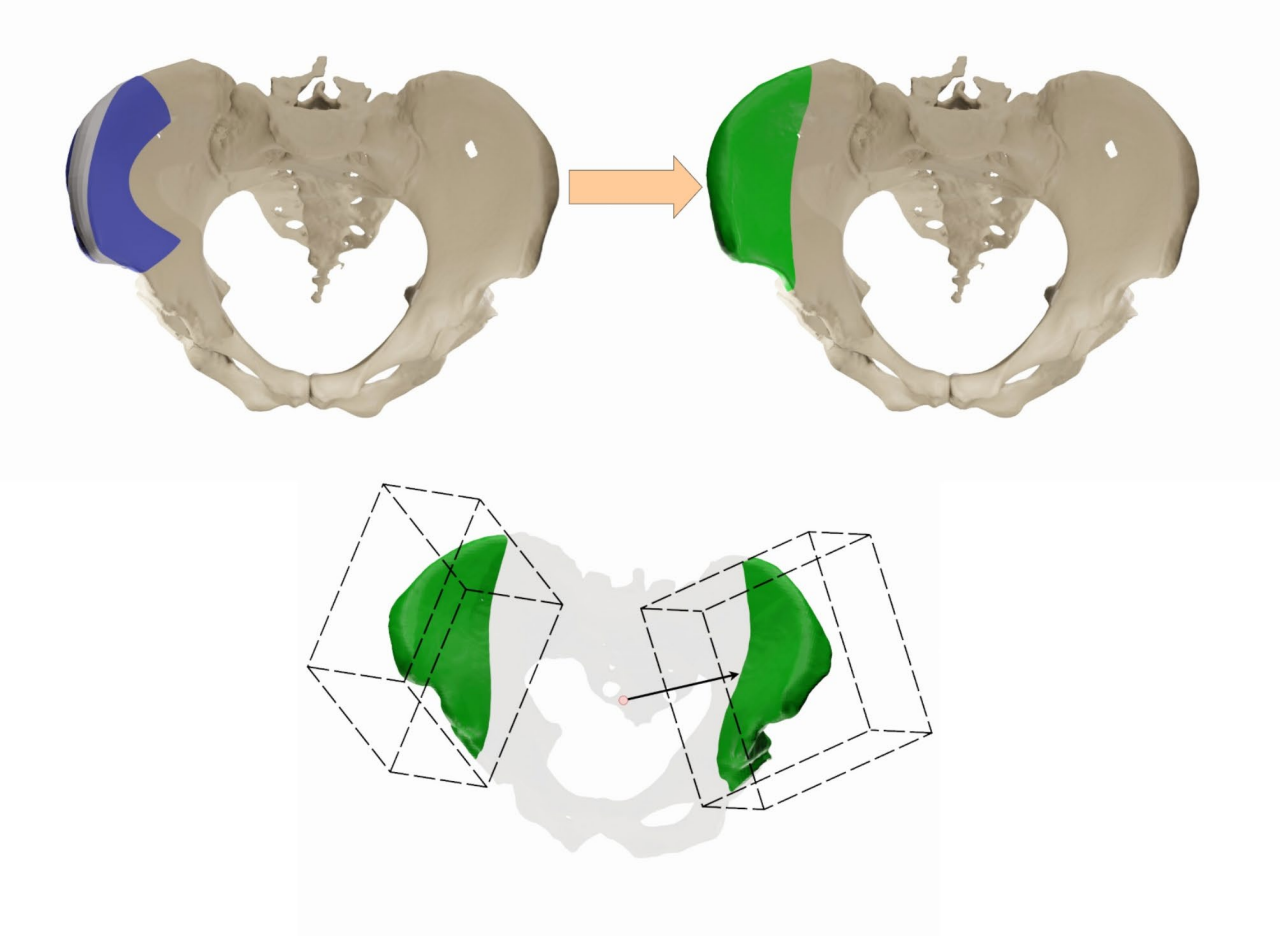
The image on the left shows the region of interest defined with the specialist assistance, colored blue (top). This geometry was then simplified by a region that is defined by a simple cutting plane, which contains all the relevant parts used in the surgical planning process. Automatically defined region of interest for transplant surgery, defined by an oblique plane in relation to the position and orientation of the pelvic bone (bottom).

Thus, a modified metric is introduced to compensate for this limitation and to focus on the relevant regions for transplant harvesting (Fig. 4). Consequently, this is the region where the segmentation needs to be as accurate as possible. The procedure of dissection is complex and involves centering the skin flap 6 cm from the anterior superior iliac spine and aligning it with an axis that joins the iliac spine to the inferior angle of the crest^42,43^. Consequently, transferring these definitions into a geometric region is a challenging task.

Within the preparation of the present work, an automated procedure to calculate the demarcation of the relevant region for transplantation was developed and applied within the present work for the first time. Thus, only the iliac crest region is effectively considered for the evaluation using these modified metrics.

The main challenge of this task was to find a generalized geometric definition of this region, which is complex and usually found by surgeons using guidelines that are difficult to express automatically for any possible pelvic bone. Thus, the solution proposed here was to find the region empirically and replicate the process for every mesh.

In consideration of experts in facial reconstruction surgery, the region was defined for pelvic bone, taking as reference the inferior iliac spine and 3 cm from the sacrum. This region was subsequently transformed into a region cut by a defined plane (Fig. 4). The spatial coordinates of this plane in relation to the geometrical center of the pelvic bone were recorded and subsequent experiments were done to replicate the cutting plane in different pelvic bones of the test set.

### Used software and hardware

The proposed approach was implemented in Python version 3.9 and Tensorflow version 2.9^44^.

All trainings and evaluations were conducted on a high-end desktop computer system with the following specifications: Processor: Intel(R) Core (TM) i9-7900X CPU @ 3.30 GHz 3.31 GHz RAM: 64.0 GB DDR4 3200 MHz Memory Graphics Card: NVIDIA GeForce RTX 2080 Ti memory size 11 GB. Operating System: Windows 10 Enterprise 64-bit.

## Results

### Qualitative results

**Fig. 5:**
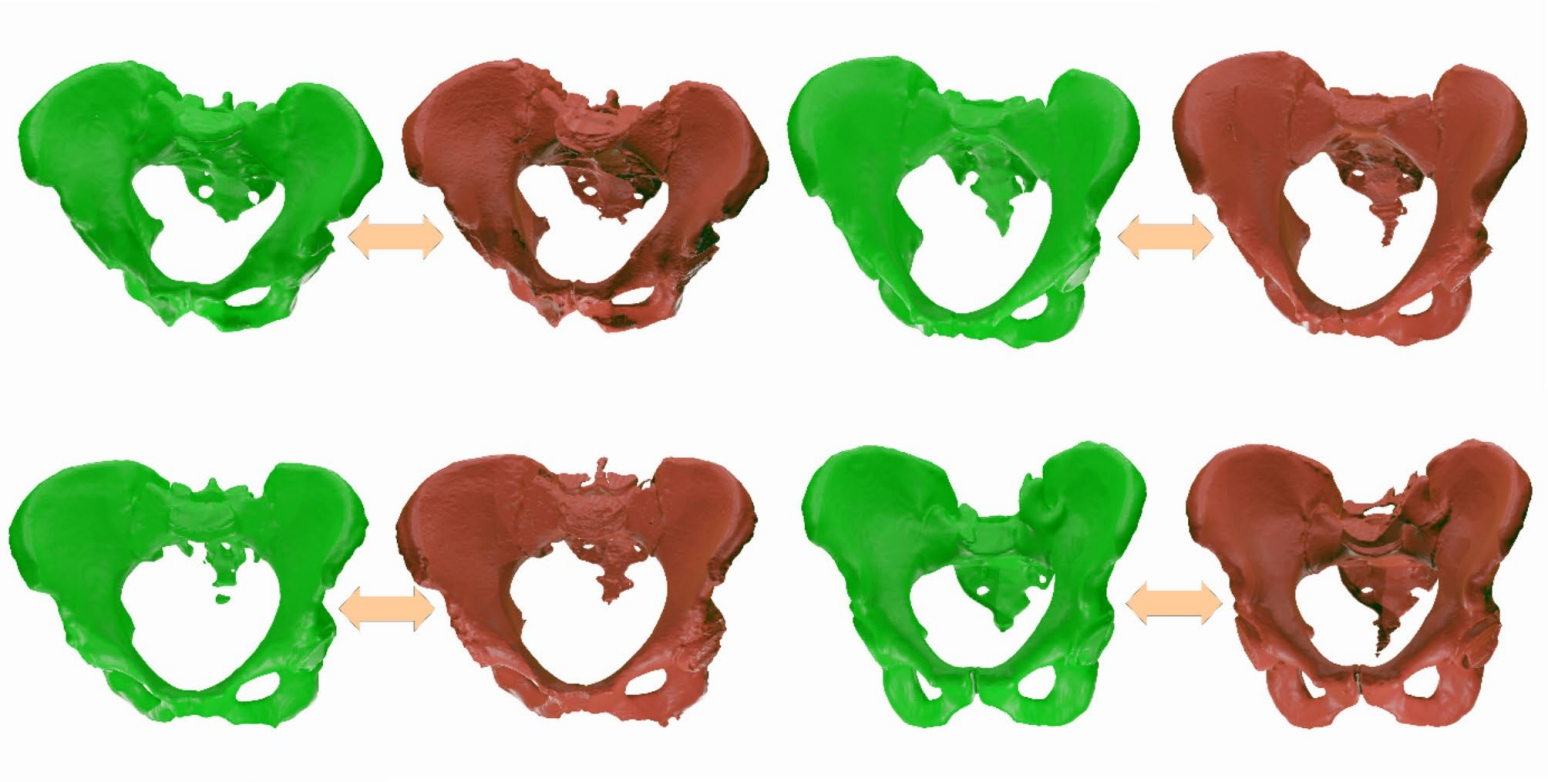
Example visualizations of pelvic bone segmentation: Predictions (green) next to corresponding ground truths (red). The optical similarity is striking, no large gaps are present in the predicted bone surfaces and no significant outliers (e.g. parts of other bones) were detected.

A few examples of prediction outputs, together with their respective ground truths, are shown in Fig. 5. A visual analysis displays the apparent high quality of the predictions, for most parts of the bone, the result is similar to the expected ground truths. Some of the known inconsistencies of ground truth meshes are also present in the predictions, i.e., the regions where the Neural Networks struggle the most to segment are perceptibly the sacrum and the coccyx.

**Fig. 6:**
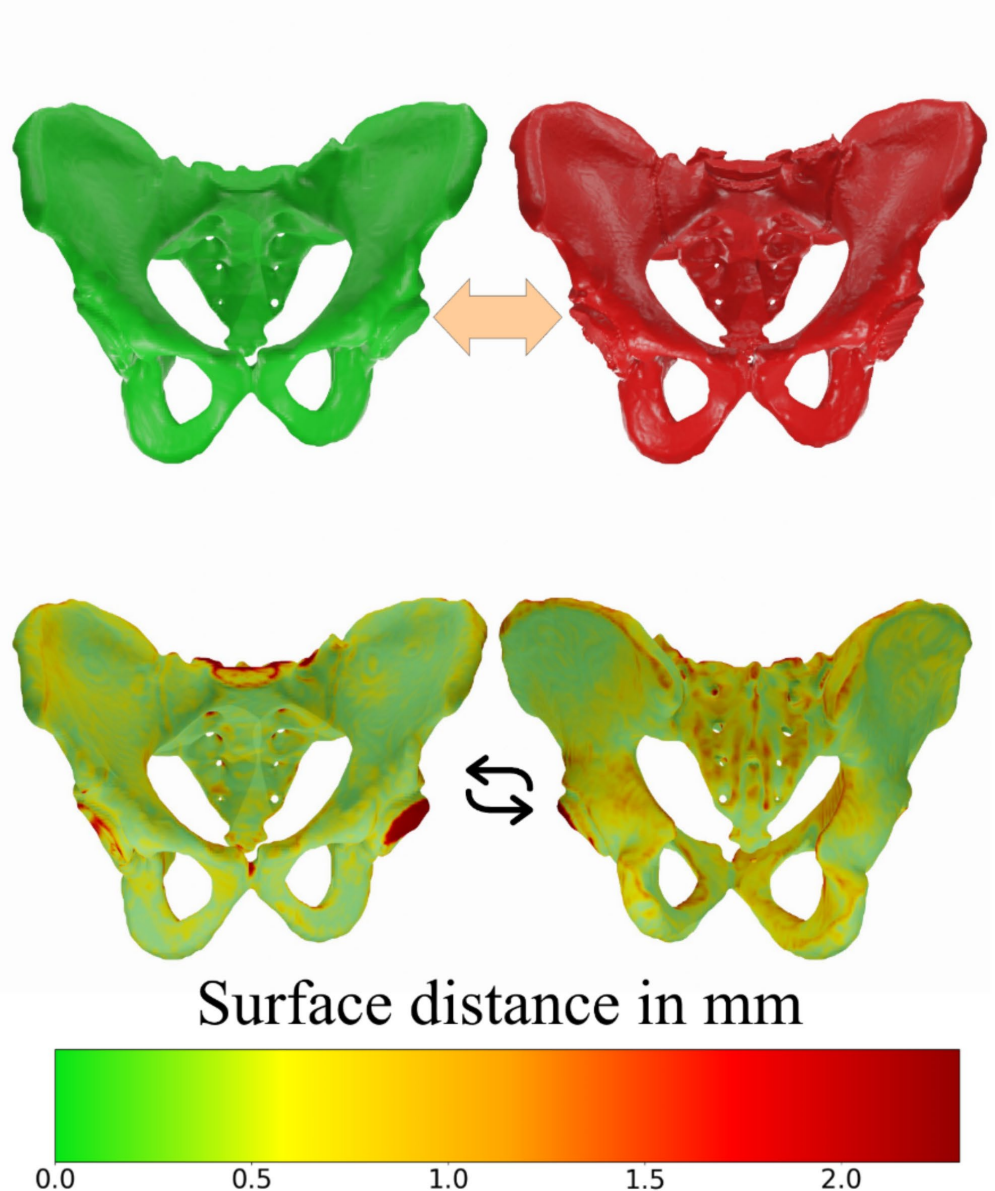
Pelvic bone segmentation: Comparison of ground truth and prediction as a color-scale display of surface distance; detailed view from anterior and posterior. Clearly visible inaccuracies are located at the cut-off femoral heads and the sacrum.

Local surface distance can be visualized by mapping a calibrated color scale on the surface of the predicted geometry (Fig. 6). This visual representation provides an overview of the quality and potentially highlights flaws of the respective predictions, which are not always clear when looking only at the numeric values of the different metrics.

**Fig. 7:**
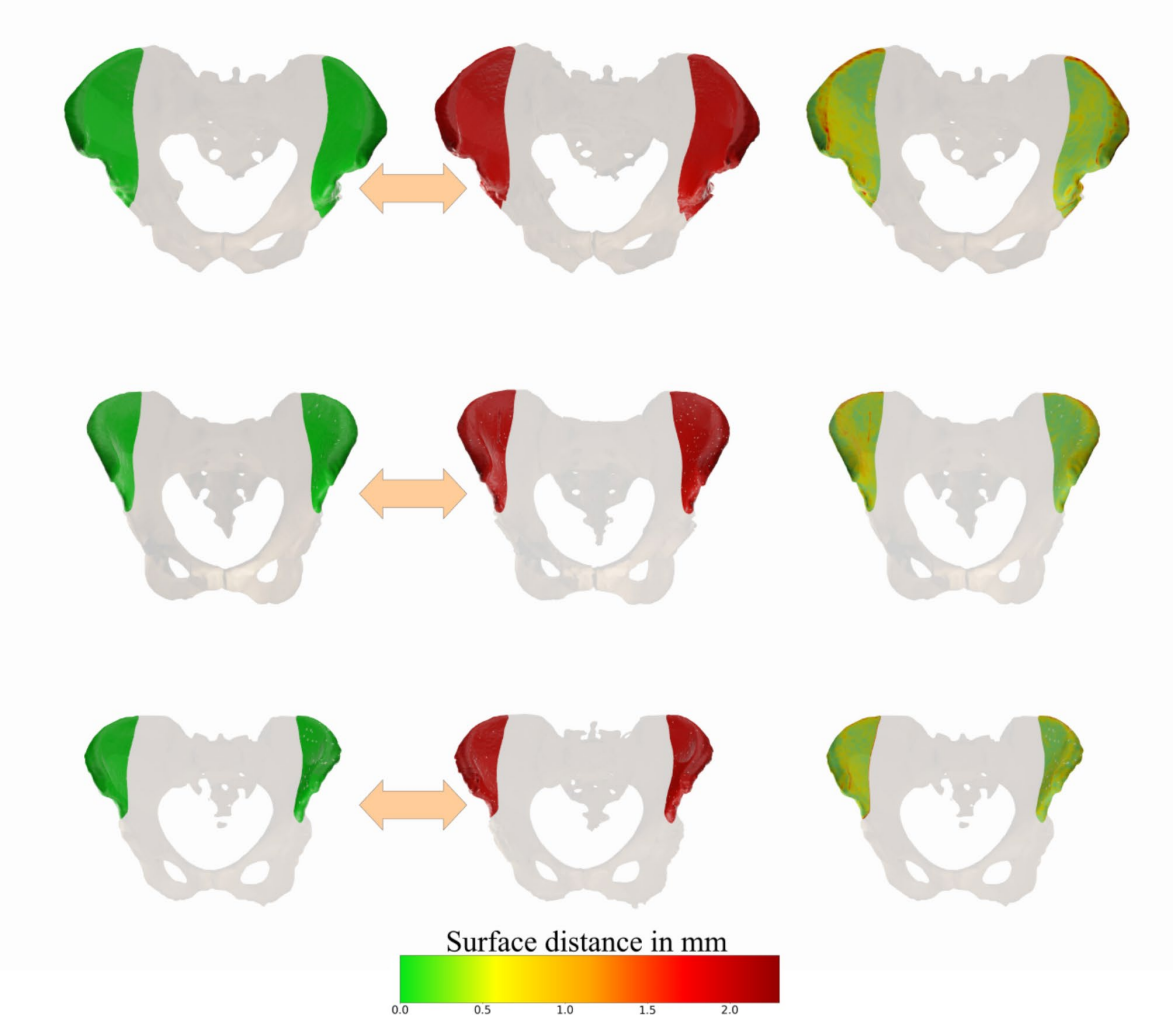
Pelvic bone segmentation limited to the automatically detected region of interest: prediction (left, green), ground truths (middle, red) and color-coded 3D surface comparison (right).

Overall, the results show that relevant parts of the pelvic bone for transplantation are well segmented, with surface distances typically within a range below 1 mm. However, there are also a few regions of the bone that have a bigger deviation. The worse segmented sections include the lumbosacral joint, and the hip joints that connect to the femur. Regarding the desired region of interest, both iliac crests for this case are well segmented, with a low deviation compared to the available ground truth. Typically, only minor sections of it have distances above 1.5 mm, which is an acceptable range. When limiting the evaluation to the relevant region of interest it is obvious that the distance values are in a particularly low range, being mostly below 0.5 mm of deviation (Fig. 7). Only some minor sections on the top have deviations close to 2 mm.

### Quantitative results

**Fig. 8:**
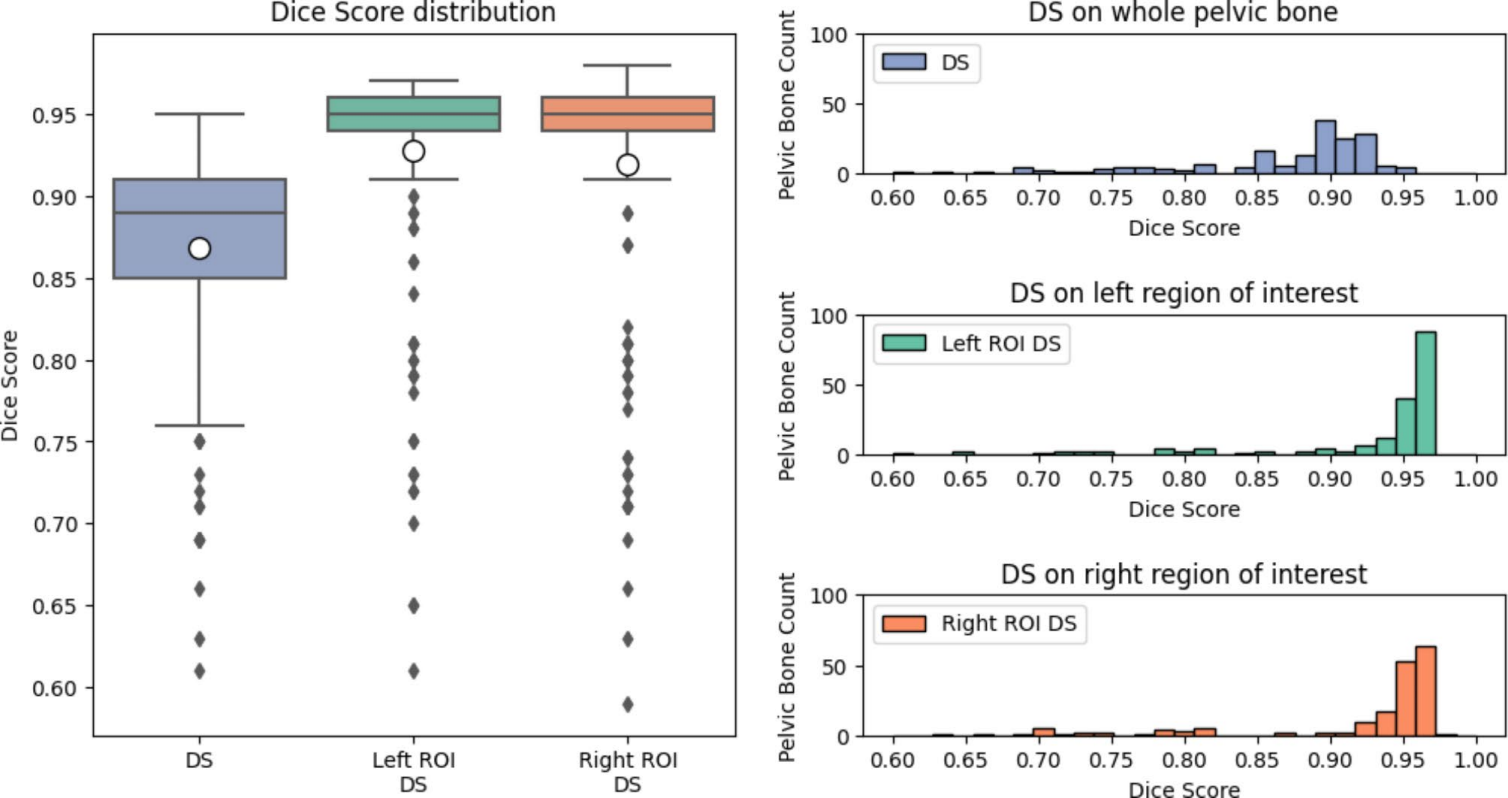
Dice score of the overall segmentation of the pelvic bone and specifically evaluated at the left and right regions of interest, respectively.

To provide a graphical visualization of the metrics evaluated on the test set, and therefore a better analysis, box plots and histograms are used. In addition to the statistics of the whole bone, the distributions for the metrics evaluated for the region of interest are also presented, both for the left and right iliac crests. The Dice score distribution can be seen in Fig. 8. The first quartile is above 0.85 together with the average, while the median is close to 0.9. When evaluating the metrics only in the region of interest, the results improve significantly.

The box plot shows that both Dice scores average for the left and right iliac crest regions are above 0.9, and there is a significantly high number of patients for which the scores are above 0.95 for both sides.

Without the restriction of the evaluation to the ROI for transplantation, which is significantly better segmented than regions irrelevant for transplant harvesting, the Dice score average is around 0.87 ± 0.07.

When evaluating the metrics on the region of interest, the results improve significantly. The box plot shows that both Dice scores average for the left and right iliac crest regions are 0.93 ± 0.07 and 0.92 ± 0.08, with a high number of cases with scores above 0.95 on both sides. There is, however, also a relevant number of outliers.

**Fig. 9:**
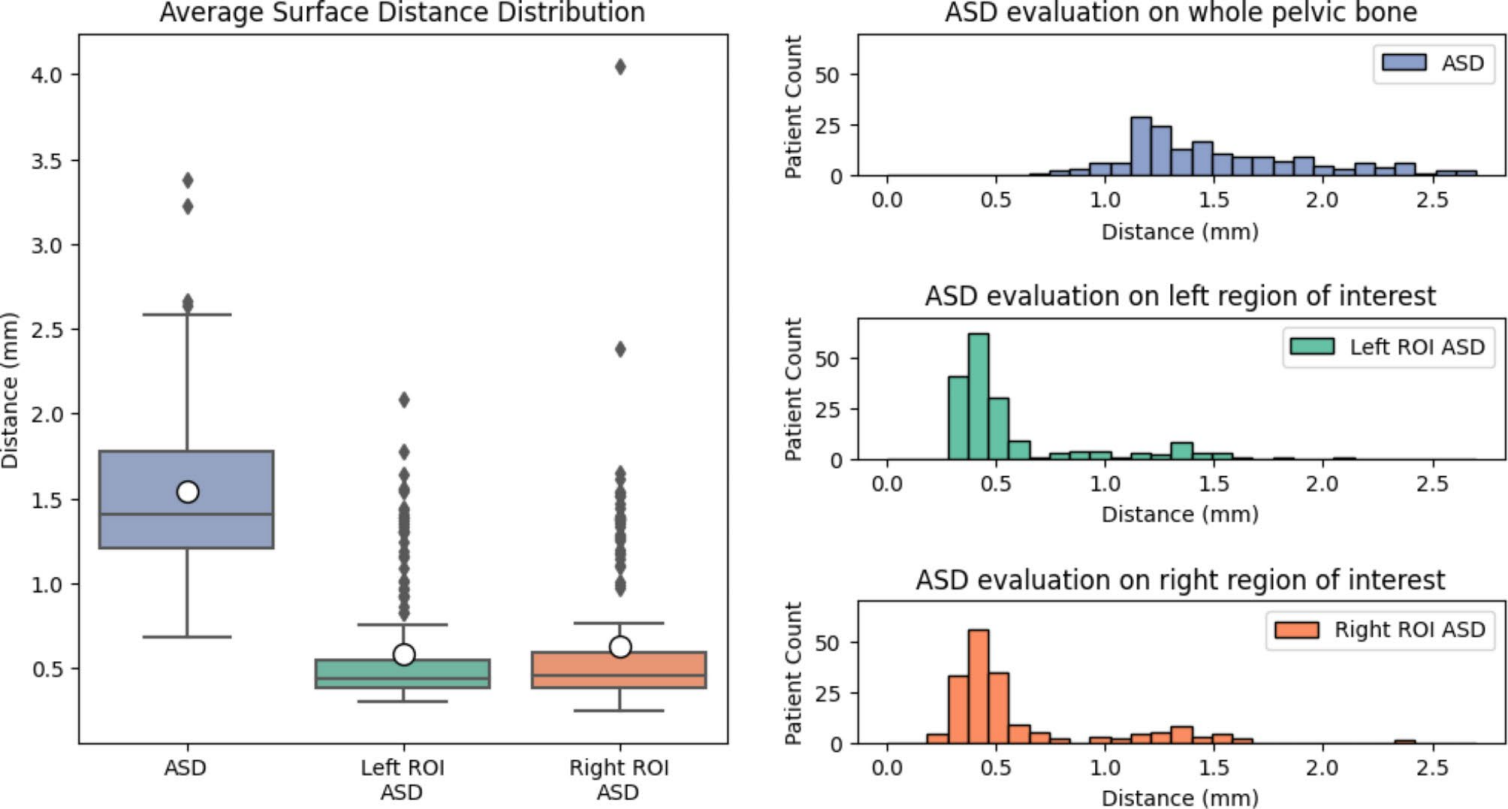
Average Surface Distance metric of comparisons between the predicted segmentation of the pelvic bone and the corresponding ground truth data for the whole bone and specifically evaluated at the left and right regions of interest, respectively.

For the Average Surface Distance (ASD) metric, statistical distributions align well with the qualitative analysis (Fig. 9). Even though the surface distance is within an acceptable range, there are several regions of the pelvic bone for which the deviation is significant, which affects the overall metrics.

The ASD evaluated on the whole pelvic bone has an average of 1.54 mm ± 0.46 mm, which can be considered a fairly accurate range, when considering the high complexity of the bone. There are also a low number of outliers, which have metrics even above 3.5 mm.

For the region of interest, the results are significantly better, showing a major improvement in the surface distance metrics, with 0.58 mm ± 0.35 mm and 0.63 mm ± 0.46 mm on the left and right ROI, respectively, as well as in the reduced number of outliers (Table 1).

As the last metric, the 95% Hausdorff Distance shows an average close to 6.5 mm, which indicates a relatively high deviation on the worst segmented regions of the bone. This result is observed in the qualitative analysis since there are a few minor regions of the bone that are poorly segmented.

**Fig. 10:**
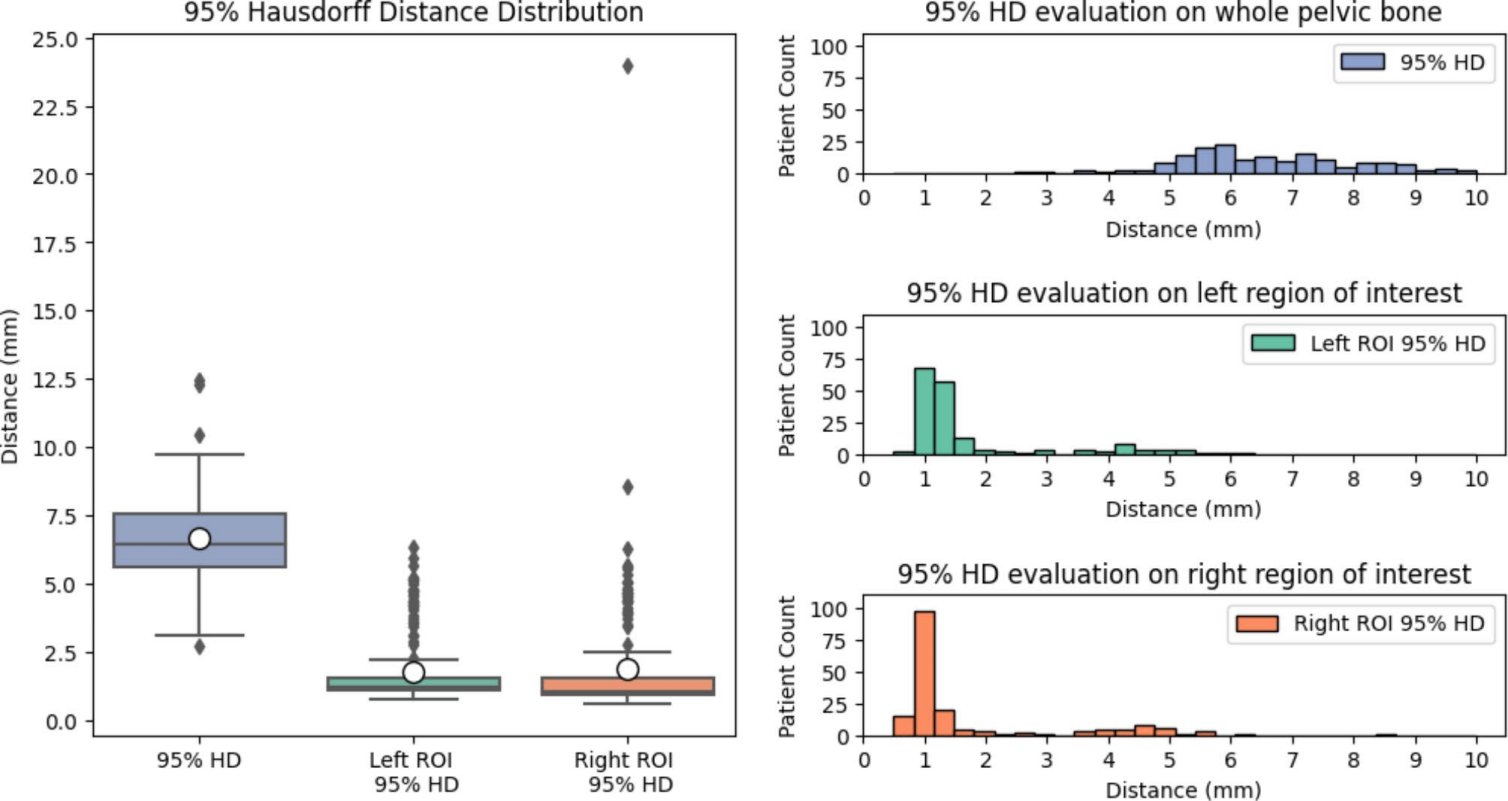
95%-Hausdorff Distance metric of comparisons between the predicted segmentation of the pelvic bone and the corresponding ground truth data for the whole bone and specifically evaluated at the left and right regions of interest, respectively.

For the regions of interest, the metrics again improve significantly, and the average for both sides is below 2 mm. Even though the box plot shows a high number of outliers, the histograms indicate that the vast majority is within an acceptable range (Fig. 10). For both iliac crests, there are more than 50% of the cases in 95% Hausdorff Distances within the range of 1 mm, which can be considered an excellent result (Table 1).

**Table 1:** Statistical data on Dice similarity coefficients (DSC), average surface distances (ASD) and 95%-Hausdorff distances (95%HD), displaying mean, standard deviation, 25% percentile, median, 75% percentile and maximal values on the whole bone as well as the corresponding left and right ROI.

		mean	std	min	25%	50%	75%	max
DSC	Whole DSC	0.87	0.07	0.61	0.85	0.89	0.91	0.95
	Left ROI DSC	0.93	0.07	0.61	0.94	0.95	0.96	0.97
	Right ROI DSC	0.92	0.08	0.59	0.94	0.95	0.96	0.98
ASD	Whole ASD	1.54	0.46	0.68	1.21	1.41	1.78	3.38
	Left ROI ASD	0.58	0.35	0.30	0.38	0.44	0.55	2.08
	Right ROI ASD	0.63	0.46	0.25	0.38	0.46	0.59	4.05
95%HD	Whole 95% HD	6.67	1.55	2.71	5.61	6.48	7.54	12.48
	Left ROI 95% HD	1.79	1.29	0.79	1.10	1.22	1.56	6.34
	Right ROI 95% HD	1.90	2.25	0.63	0.93	1.08	1.57	23.99

## Discussion

The geometry of the pelvic bone is considerably more complex than that of many other bones. Besides its relatively large dimensions, it consists of thin cortical layers and large spongy compartments of different apparent densities, which complicate the task of automated bone segmentation. Until now, there are only few published works that studied the segmentation of the pelvic bone, both with statistical shape models and methods based on Neural Network.

The qualitative analysis showed high accuracy in the segmentation of the bone for most regions of the pelvis. While there are some sections with visually high deviations, which are especially concentrated on the coccyx, the femoral joints and the spine, most of the iliac crest seems to be well segmented for most cases, which is the region used for the surgical procedure.

Additionally, a region that is typically poorly segmented is the hip joint, that unites the pelvic bone with the femur. This is an expected result, because this region is usually cropped carelessly on the available so-called ground truth data, since it is a region of no interest for the surgery. Therefore, the Neural Networks struggle to provide accurate segmentation to this region, which affects considerably the evaluated metrics.

Visual analysis showed high accuracy of the segmentation, especially for the region of interest, outcomes that indicate that, even though the framework is not perfect to segment all regions of the bone, it does an especially good in segmenting the donor site that is relevant for subsequent planning of reconstructive surgery of the facial bones.

These findings are confirmed in the quantitative analysis, which shows excellent results and a significant improvement on all three metrics (dice similarity coefficient, average surface distance and 95%-Hausdorff-Distance) when evaluated focusing on the region of interest, rather than in the whole bone. These results are especially outstanding when considering the complexity of the bony geometry of the pelvic ring and the locally variable apparent densities.

Thus, the proposed method to segment the pelvic bones provides overall good results, especially on the iliac crest, which is used in the facial reconstructive surgery. However, when applying this segmentation approach, additional caution has to be taken, since for a low number of cases, the outcomes are not suited for the surgical planning, and possible manual post-processing procedures would be necessary.

The proposed method has also known limitations, with a few examples for which the output segmentations are problematic for the desired application. These findings refer to bones that have evaluation metrics significantly worse than most of the bones. However, they are still statistically relevant for the study since it is also important to evaluate the robustness of the model.

This work provides novel contributions to the medical image segmentation field as a whole. All the techniques proposed throughout this work could potentially be applied to other applications, and even to segment other types of images.

Another main contribution is the introduction of a problem-specific metric. It is not rare to find cases in medical image segmentation tasks where the standard evaluation metrics fail to provide clinically relevant results. Some regions in images to be segmented are more relevant than others, and metrics that give the same importance to all the regions are poor in explaining the accuracy of the metrics.

There has been growing research for possibilities to improve facial reconstructive surgery planning procedure^11,45,46^. The use of computer-assisted tools in the pre-operative process has been deeply investigated and has proved to provide several advantages to the surgery^9,10^.

However, the main source of limitation on the planning procedure, when using computer tools, is the segmentation of the bones of interest from CT imaging^17^. The process done in the present day is costly, time-consuming, and completely non-reproducible since it is done based on threshold segmentation and a highly manual post-processing step.

Regarding the pelvic bone, some attempts of segmenting this highly complex bone have been made^25–27^, yet no works are analyzing the possibility of an automatic pipeline to segment this bone as a donor site for the facial reconstructive surgery. Thus, the present work provides not just a fully working pipeline to automatically segment this bone, but also a specific metric to evaluate the precision of the results in the region of interest for transplant surgery.

Additionally, qualitative results showed a high accuracy on those segmentations for the iliac crests, which is the relevant part for transplant harvesting. The average surface distance metric evaluated on these regions showed values below 0.63 mm and 0.58 mm for both sides, which is within a range that would allow for further utilization on real planning procedures^8^.

These results are not directly comparable to the ones found in the literature for the segmentation of the pelvic bone. This is since even though the proposed approach achieves significantly better metrics than Liu et al.^26^, having a better Dice score for the whole bone, for instance, the nature of both datasets is different. While this work was conducted using solely CT images and ground truths based on this data, in the work by Liu et al. MRI data was used.

A comparison with a deep learning-based method introduced by Liu et al.^27^ for the application on the pelvic bone indicates highly accurate results, as they could achieve a high Dice score of 0.99 for the whole pelvic bone in their work. For the whole bone, the present experiments reach a comparatively low value of 0.87 +- 0.07, with presumably higher inter- and intra-observer variations at the stage of generation of the so-called ground truth data of the present study. As mentioned above, there is a high variability in the quality of the data used to perform the training on the present work, both in the quality of the raw CT images as well as in the ground truth segmentations. The irregularity on the ground truths can be explained by the fact that they are only carefully segmented on the region that is relevant for the surgery procedure, and thus regions like the sacrum and coccyx are segmented worse for a high number of patients on the set.

In problematic cases, i.e. showing poor values in the above metrics, flaws are especially present in the coccyx, the connections toward the femur and the top region toward the spine. However, other sections like around the iliac crest are also not always perfectly well segmented. As the region of most interest for the desired application is the iliac crest these cases may be problematic for further use in reconstructive surgery.

Another important remark about the work conducted is the presence of a significant number of outliers in the evaluation results. In the set, there are a few cases where the metrics attest poor segmentations on the region of interest, attesting, for instance, average surface distance values of above 1.5 mm, and 95% Hausdorff Distance values of over 4 mm. In future work, it is intended to include the more difficult cases, e.g. with implants, that were intentionally left out in the present study, by the defined exclusion criteria, in order to investigate the model’s capability of performing segmentations in these cases that are closer to a comprehensive clinical application.

It is important to note that this does not hinder the use of this method in surgical planning scenarios. Firstly, this happens only for a small portion of cases in the set, e.g. the ASD values are over 1.5 mm for less than 4% of the patients. Secondly, the method could potentially be followed by a corrective manual post-processing step in cases where the segmentation is not sufficient.

One limitation of the proposed approach may be the specific definition of the regions of interest. As explained above, the method of defining the ROI at the iliac crests is based on a fully automated procedure that was heuristically defined according to a schematic guideline and may therefore vary between different bones in respect to its actual anatomical shape. However, this does not seem to be a problem since careful revision of the defined regions showed a correct placement and dimensioning of the ROI.

Hybrid loss functions, which combine multiple terms to handle class imbalance, have shown promising results in improving segmentation outcomes^47,48^. While Tversky loss was most effective in our context, exploring hybrid approaches could further enhance accuracy and robustness, making this a potential avenue for future work. Furthermore, attention gate mechanisms have been proposed for different tasks in medical image segmentation and shown their capabilities to enhance segmentation accuracy by focusing on salient features and suppressing irrelevant regions^49^. This approach may be evaluated in future work to explore the potential for more compact and efficient single-stage network architecture for pelvic segmentation.

## Conclusion

The approach presented in this work with a two-stage 3D U-Net proved successful in extracting individual iliac crest geometries from CT data. Thus, it has the potential to serve as an essential starting point in a digitized planning process, providing data for subsequent surgical planning. Through the complete automation of the segmentation step, the entire patient-specific planning can not only be significantly expedited but also be made reproducible and, therefore, the surgical planning becomes objective, while maintaining its patient specific focus.

## Data Availability

The raw medical imaging datasets analyzed during this study are not publicly available due to privacy concerns, as per the guidelines of the ethics committee, which permits access to the original data only for a specific group of named researchers. However, evaluation data and derived statistical analyses are available from the corresponding author upon reasonable request.
